# Isolation of Fungi from a Textile Industry Effluent and the Screening of Their Potential to Degrade Industrial Dyes

**DOI:** 10.3390/jof7100805

**Published:** 2021-09-27

**Authors:** Juvenal Juárez-Hernández, Dalia Castillo-Hernández, Cristhian Pérez-Parada, Soley Nava-Galicia, Jaime Alioscha Cuervo-Parra, Edy Surian-Cruz, Gerardo Díaz-Godínez, Carmen Sánchez, Martha Bibbins-Martínez

**Affiliations:** 1 Centro de Investigación en Biotecnología Aplicada, Instituto Politécnico Nacional, Tepetitla de Lardizabal 90700, Tlaxcala, Mexico; juvenal1a@hotmail.com (J.J.-H.); dcastillohe@ipn.mx (D.C.-H.); hydeist_090487@hotmail.com (C.P.-P.); snava@ipn.mx (S.N.-G.); suce24@hotmail.com (E.S.-C.); 2 Escuela Superior de Apan, Universidad Autónoma del Estado de Hidalgo, Carretera Apan-Calpulalpan, Chimalpa Tlalayote, Apan 43900, Hidalgo, Mexico; alioscha@uaeh.edu.mx; 3 Laboratorio de Biotecnología, Centro de Investigación en Ciencias Biológicas, Universidad Autónoma de Tlaxcala, Tlaxcala 90120, Tlaxcala, Mexico; diazgdo@hotmail.com (G.D.-G.); carmen.sanchezh@uatx.mx (C.S.)

**Keywords:** *Emmia latemarginata*, biodegradation, decolorization, industrial dyes, textile effluent, ligninolytic enzymes

## Abstract

Six fungal strains were isolated from the textile industry effluent in which they naturally occur. Subsequently, the fungal strains were identified and characterized in order to establish their potential decolorizing effect on textile industry effluents. The strains of interest were selected based on their capacity to decolorize azo, indigo, and anthraquinone dyes. Three of the strains were identified as *Emmia latemarginata* (MAP03, MAP04, and MAP05) and the other three as *Mucor circinelloides* (MAP01, MAP02, and MAP06), while the efficiency of their decolorization of the dyes was determined on agar plate and in liquid fermentation. All the strains co-metabolized the dyes of interest, generating different levels of dye decolorization. Plate screening for lignin-degrading enzymes showed that the MAP03, MAP04, and MAP05 strains were positive for laccase and the MAP01, MAP02, and MAP06 strains for tyrosinase, while all strains were positive for peroxidase. Based on its decolorization capacity, the *Emmia latemarginata* (MAP03) strain was selected for the further characterization of its growth kinetics and ligninolytic enzyme production in submerged fermentation under both enzyme induction conditions, involving the addition of Acetyl yellow G (AYG) dye or wheat straw extract, and no-induction condition. The induction conditions promoted a clear inductive effect in all of the ligninolytic enzymes analyzed. The highest level of induced enzyme production was observed with the AYG dye fermentation, corresponding to versatile peroxidase (VP), manganese peroxidase (MnP), and lignin peroxidase (LiP). The present study can be considered the first analysis of the ligninolytic enzyme system of *Emmia latemarginata* in submerged fermentation under different conditions. Depending on the results of further research, the fungal strains analyzed in the present research may be candidates for further biotechnological research on the decontamination of industrial effluents.

## 1. Introduction

Globally, at least 10% of the total textile dye produced (approximately 280,000 tons) is discharged in industrial effluent every year [1,2], with the colored wastewater produced by the textile industry rated as the most polluted wastewater of all industrial sectors. It has been estimated that over 10,000 different textile dyes and pigments are in common use, while total world organic colorant production is estimated at more than 100,000 tons per year [3,4]. Synthetic textile dyes are one of the most dangerous pollutants [5]. Based on the chemical structure of the chromophore group, dyes can be classified as azo, anthraquinone, triphenylmethane, phthalocyanine, or polyaromatic. Most are mutagenic and/or carcinogenic and, moreover, their removal from the environment is very difficult. The decolorization of textile wastewater is still a major environmental concern because of the difficulty of removing synthetic dyes via conventional treatment systems [6,7,8,9,10]. Physico-chemical treatment methods (adsorption and precipitation methods, chemical degradation, or photodegradation) are economically and often methodologically demanding, as well as being time-consuming and largely ineffective. However, there is growing preference, in general terms, for the application of modern biological techniques to tackle different environmental problems. As biological remediation is considered more efficient in terms of its long-lasting benefits and minimal harmful effects on the environment, conducting biodegradation via the use of different microorganisms appears to be an alternative [11,12]. Although many bacteria are able to degrade synthetic dyes under anaerobic conditions, in these circumstances the dyes are usually reduced to even more toxic aromatic amines. Therefore, the ability of filamentous fungi to degrade toxic compounds, including synthetic dyes, has been widely studied as has their potential as agents of biodegradation [13,14,15,16,17,18,19]. Within this group of fungi, Basidiomycetes, especially white-rot fungi, are the most intensively studied group of organisms in terms of their ligninolytic and bioremediation abilities. Fungi such as *Phanerochaete chrysosporium*, *Pleurotus ostreatus*, *Bjerkandera adusta*, and *Trametes versicolor* have been intensively studied in the context of the decolorization processes [20,21,22,23,24]. Their enzymatic system, in which enzymes participate in the modification of lignin (laccase, lignin peroxidase, manganese peroxidase, and H_2_O_2_-producing oxidases etc.), is able to transform different xenobiotic compounds, such as polycyclic aromatic hydrocarbons, polychlorinated biphenyls, pesticides, or synthetic dyes [25,26,27].

The present research studied the decolorization potential of fungal strains, isolated from a textile industry effluent in which they naturally occur, for use on reactive and disperse synthetic dyes belonging to different chemical groups. The screening conducted found six strains that presented different decolorization properties, including some *Emmia latermarginata* and *Mucor circinelloides* strains that have, to date, attracted little interest in the literature. Given the dye decolorization results presented here, these strains could be candidates for further biotechnological research on the decontamination of industrial effluents.

## 2. Materials and Methods

### 2.1. Chemicals

All of the industrial textile dyes used—disperse (black, yellow, and red), Remazol (yellow, red, and blue), and indigo—were obtained from the industrial textile company Parras S.A. de C.V. (Industrial Zone 2000, Puebla, Mexico), with the exception of the Acetyl Yellow G (AYG) dye (Sigma-Aldrich, St. Louis, Mo, USA) (Table 1).

### 2.2. Culture Conditions and Isolation of Fungi from a Textile Industry Effluent

Fifty samples were taken from the colored effluent produced by the textile facility and then placed separately in sterile 125 mL flasks. The samples were incubated for 15 days at room temperature, with serial dilutions in sterile water then carried out. Petri dishes containing Saboraud dextrose agar (SDA, NEOGEN^®^, Lansing, MI, USA) were inoculated with 500 µL of each dilution and incubated for up to 30 days at 28 °C. The colonies isolated from the samples were classified based on morphological parameters, isolated on individual plates, and if necessary, purified by means of a series of plate transfers. The isolates were kept in mycosel agar (Bioxon^®^, Mexico City, Mexico), to which 500 mg^−1^ of ampicillin (Sigma-Aldrich) was then added to avoid bacterial contamination. All isolates were grown on a medium containing 15 g L^−1^ of malt extract agar (MEA, Fluka, Madrid, ESP), 3 g L^−1^ of starch, and 20 gL^−1^ of agar slants in screw-cap tubes kept at 4 °C [28].

### 2.3. Fungal Strains Identification

The molecular identification of the fungal isolates was carried out using the internal transcribed spacer (ITS) region of ribosomal DNA (rDNA), while restriction fragment length polymorphism (RFLP) analysis was conducted on the ITS regions of the rDNA with random amplified polymorphic DNA (RAPD) analysis then undertaken on the total genomic DNA.

#### 2.3.1. Genomic DNA Extraction and PCR Conditions

The genomic DNA extraction involved growing each pure isolate in 150 mL nutritive broth (NB) for 17 days at 30 °C, under 120 rpm shaking conditions. Each flask was inoculated with five mycelial plugs taken from the periphery of each fungal colony and grown for 7 d at 30 °C in Petri dishes containing potato dextrose agar (PDA, Bioxon). For the DNA extraction, approximately 100 mg of fungal mycelia was homogenized in a clean and autoclaved mortar and pestle with liquid nitrogen, with the homogenate then purified using the ChargeSwitch gDNA Plant Kit (Invitrogen, Waltham, MA, USA), following the manufacturer’s protocol and recommendations for the purification of the total genomic DNA of the fungi. The concentration of purified DNA samples was measured at 260 nm absorbance using a NanoDrop-1000 spectrophotometer (NanoDrop Technology, Rockland, DE, USA) and stored at −20 °C until use.

#### 2.3.2. PCR Amplification

The ITS region of the rRNA gene was amplified using a previously described forward ITS5 (52032-GGAAGTAAAAGTCGTAACAAGG-3′) and reverse ITS4B (5′-TCCTCCGCTTATTGATATGC-3′) primer set [29,30] under the following PCR conditions. The 50 μL PCR reaction conducted used 1.5 U Taq DNA polymerase (Biotools, Madrid, Spain), 1 μM of each primer, 0.1 mM DNTPs, 1.25 mM MgCl_2_, 1X Buffer Taq, and 200 ng genomic DNA as a template, all of which were diluted in PCR grade water.

The PCR amplification reaction was performed in a Bio-Rad thermal cycler with an initial denaturation of 4 min at 95 °C, followed by 30 cycles at 95 °C for 30 s, 55 °C for 30 s, and 72 °C for 1 min, while a final extension was conducted at 72 °C for 10 min. The PCR products were analyzed on 1.5% agarose gels containing ethidium bromide, purified using the ChargeSwitch^®^-Pro PCR Clean-up Kit (Invitrogen) to remove unincorporated primers and nucleotides, and then subjected to electrophoresis on agarose gels.

The samples were cloned in the CloneJET ^TM^ PCR Cloning Kit (Fermentas, Waltham, MA, USA), for further RFLP analysis, or sent to the sequencing service at the Biotechnology Institute of the Universidad Nacional Autónoma de México (National Autonomous University of Mexico) for sequencing. Each sequencing reaction used 0.5 mM of ITS5 primer and 100 ngL^−1^ of the purified PCR fragment.

The sequences were analyzed using Sequence Scanner Version 1.0 (Applied Biosystems, Waltham, MA, USA), Bioedit, Serial Cloner, and Clustal W and compared with previously published sequences held in public databases, using the BLASTN program for species identification (http://blast.ncbi.nlm.nih.gov/Blast.cgi, accessed on 21 July 2021). The phylogenetic analysis of the sequences aligned with the *Emmia latemarginata* and *Mucor circinelloides* strains was performed for the ITS region of the rDNA by generating a neighbor-joining tree with the Kimura two-parameter model, supported by the bootstrap method and using 1000 random replicates with the MEGA X software [31].

#### 2.3.3. Restriction Analysis

The ITS sequences from the fungal isolates were first aligned with sequences from NCBI database. The analysis identified two main strains groups, Group A (MAP03, MAP04, and MAP05) and Group B (MAP01, MAP02, and MAP06). Restriction patterns of the ITS sequences were predicted using pDRAW32 and Serial Cloner 1.2. Predicted restriction fragments were compared to choose the best discrimination. Finally, two restriction endonucleases for each group were selected, enzymes *Sal I* and *Alu I* for Group A and the enzymes *HindIII* and *MboI* for Group B. Digestion was performed by incubating a 5 μL aliquot of PCR product with 10 U of the respective enzyme in a final volume of 20 μL at 37 °C for 2 h. After the electrophoretic separation, which was conducted on 2.5% agarose gels containing ethidium bromide, the images were captured using the GelDoc documentation system (Bio-Rad^®^, Hercules, CA, USA).

#### 2.3.4. RAPD Analysis

The RAPD analysis undertaken used the Ready-To-Go RAPD kit (GE Healthcare, Bucks, UK) with solely the RAPD Primers 2 (5′-d[GTTTCGCTCC]-3′), 5 (5′-d[AACGCGCAAC]-3′), and 6 (5′-d[CCCGTCAGCA]-3′) employed under the PCR conditions of an initial denaturation for 5 min at 95 °C, followed by 45 cycles at 95 °C for 1 min, 36 °C for 1 min, and 72 °C for 2 min. The final elongation step was increased to 10 min, after which the samples were cooled to 4 °C. The PCR products were analyzed on 2.5% agarose gels containing ethidium bromide, while gel images were captured using the GelDoc documentation system (Bio-Rad^®^) and the band profile was analyzed for the further generation of phylogenetic trees using the Quantity One 4.6.3 program (BIO-RAD^®^), scoring 1 for the presence of major bands and 0 for their absence.

### 2.4. Fungal Characterization

#### 2.4.1. Dye Decolorization on Agar Plate

Two different culture media were prepared: (1) A medium containing 20 g L^−1^ agar (Sigma-Aldrich, St. Louis, Mo, USA) and 150 mg L^−1^ dye; and (2) A medium containing 20 g L^−1^ agar, 3.5 g^−1^ MEA (malt extract agar, Sigma-Aldrich, St. Louis, Mo, USA), 10 g L^−1^ starch (Merck-Millipore, Burlington, MA, USA), and 150 mg L^−1^ dye. The culture media were autoclaved at 120 °C for 15 min, with the Petri dishes containing either culture medium then inoculated with 0.5 cm^2^ mycelia plugs taken from the periphery of a colony growing on SDA at 30 °C. The inoculum was placed, mycelium facing down, on the center of the culture medium, with the cultures then incubated at 28 °C for six days. The radial growth was registered daily from the second to the sixth day of incubation.

#### 2.4.2. Dye Decolorization in a Carbon-Limited Liquid System

A carbon-limited liquid medium, modified from that proposed by Janshekar and Fiechter [32], was prepared, consisting of the following, as expressed in g L^−1^: D-glucose, 3 g; ammonium tartrate, 0.66 g; MgSO_4_·7H_2_O, 0.15 g; CaCl_2_·2H_2_O, 30 mg; FeSO_4_·7H_2_O, 5.55 mg; and, 2N H_3_PO_4_, 3.27 mL. Subsequently, 150 mg L^−1^ of each dye was added separately to the culture medium, with the final pH adjusted to 4.5 using NaOH or H_2_SO_4_ solutions and the 125 mL flasks containing 80 mL of culture medium then inoculated with 2 mL of a solution containing 10^5^ spores mL^−1^ and incubated at room temperature on an orbital shaker at 150 rpm for up to 30 days. All experiments were performed in triplicate, with 1 mL of each sample taken separately every eight days and centrifuged at 2000 rpm for 5 min. The decolorization percentage was determined spectrophotometrically (using Equation (1)) at the maximum absorption point for each dye

(1) $$\mathrm{decolorization}\;(\%)=\frac{A_{\lambda },\mathrm{initial}-A_{\lambda },\mathrm{final}}{A_{\lambda },\mathrm{initial}}\times 100$$

where *A_λ_*, initial = initial absorbance and *A_λ_*, final = absorbance after days of growth.

#### 2.4.3. Plate Screening for Lignin-Degrading Enzymes

The isolated microorganisms were subjected to different plate assays to enable screening for several lignin-degrading enzymes (phenoloxidase, laccase, tyrosinase, and peroxidase). Phenoloxidase was detected via the inoculation of MEA supplemented with 0.5% (*w/v*) tannic acid, which was autoclaved separately before being added to the culture media. After 5–7 days of the culture, the brown color observed around the growth indicated positive results [33].

The tests for laccase, tyrosinase, and peroxidase were performed according to a modification of the method proposed by López et al. and Kiiskinen et al. [14,33] and then read after two to three drops of specific reagents were added to the fungal colonies grown on MEA for two and five days, respectively. For laccase, o-anisidine and guaiacol were used separately, whereby the addition of guaiacol (1.24 g in 100 mL of 96% ethanol) and o-anisidine (100 mg L^−1^, solely added to the media prior to autoclaving) obtained a purple color after more than 4 h of contact with the colonies. The addition of *p*-cresol (1.08 g in 100 mL 96% ethanol) generated an orange-brown color in the colonies showing tyrosinase activity, while the addition of equal parts of 0.4% (*v/v*) H_2_O_2_ and 1% pyrogallol in water obtained a yellow-brown color in the colonies with peroxidase activity.

### 2.5. Growth and Ligninolytic Enzyme Production of the Emmia Latemarginata (MAP03) Strain in Submerged Fermentation under Different Conditions

Four fermentations were conducted, in triplicate, with the first conducted in basal medium, the second using 500 ppm Acetyl Yellow G dye (AYG, Sigma-Aldrich), the third using 10% *v/v* wheat straw extract, and the fourth using both compounds (AYG+ wheat straw extract). The fermentations were performed in 125 mL Erlenmeyer flasks containing 50 mL of basal medium of the following composition (g L^−1^): yeast extract, 5; glucose, 10; K_2_HPO_4_, 0.4; ZnSO_4_·7H_2_O, 0.001; KH_2_PO_4_, 0.6; FeSO_4_·7H_2_O, 0.05; MnSO_4_·H_2_O, 0.05; MgSO_4_·7H_2_O, 0.5; and CuSO_4_·7H_2_O, 0.25 [34]. Mycelial plugs (4 mm diameter) taken from the periphery of *Emmia latemarginata* (MAP03) colonies grown for seven days at 30 °C in Petri dishes containing PDA were used as inoculum. The cultures were incubated at 30 °C for 22 days on a rotary shaker at 120 rpm, with three flasks taken as samples every 24 h after the second day of the fermentation process.

The enzymatic extract was obtained by filtering the cultures using filter paper (Whatman, Maidstone, UK) and then stored at −20 °C until the analysis was carried out, while the mycelium was rinsed with 0.9% NaCl and its biomass (X) determined as the difference of dry weight (g L^−1^). The biomass X = X(t) assay was conducted using the Verhulst-Pearl logistic equation [35].

The wheat straw extract used as a ligninolytic enzyme-inducer was prepared by soaking 100 g of dry wheat straw in 1000 mL of an ethanolic distilled water mixture (50% *v/v*), which was then shaken on an orbital shaker at 150 rpm for 24 h at 25 °C. After this extraction period, the straw was eliminated by filtration through a Miracloth (Merck Millipore International, Burlington, MA, USA), with the filtrate centrifuged at 4500 rpm for 10 min at 20 °C after which the supernatant was evaporated in a rotary evaporator at 76 °C for 5 h and sterilized at 121 °C for 20 min. The extract was kept frozen until use. For the induction experiments, 50 mL of the inducer extract was added to the experimental cultures described above.

### 2.6. Ligninolytic Enzyme Assays

The laccase (Lac) activity was determined by measuring changes in absorbance at 468 nm via the extinction coefficient μL _468_ = 35,645 M^−1^cm^−1^, using 2,6-dimethoxyphenol (DMP) (Sigma-Aldrich) as the substrate. The assay mixture contained 950 μL substrate (2 mM DMP in 0.1 M phosphate buffer at pH 6.5) and 50 μL enzyme extract and was incubated at 40 °C for 1 min [34].

The lignin peroxidase (LiP) activity was determined, using the veratryl alcohol oxidation assay modified from Ten Have et al. [36], by recording the increase in the absorbance at 568 nm via the extinction coefficient ε_568_ = 9300 M^−1^ cm^−1^. The assay mixture contained 950 μL substrate (2 mM veratryl alcohol dissolved in sodium tartrate buffer 0.1 M, pH 3.0), 20 μL enzyme extract, and 30 µL H_2_O_2_ 0.5 mM incubated at 35 °C for 1 min.

The manganese peroxidase (MnP) assay applied was modified from Giardina et al. [37], using MnSO_4_ as a substrate, while the assay mixture contained 950 µL substrate (0.5 mM MnSO_4_, 0.05 mM H_2_O_2_, and 50 mM sodium malonate buffer, pH 4.5) and 50 µL enzyme extract. The oxidation of Mn^2+^ to Mn^3+^ was followed by an absorbance increase at 270 nm (ε = 11,590 M^−1^ cm^−1^).

The determination of the dye peroxidase (DyP) activity used a method modified from the work of Salvachua et al. [38], following the oxidation of 2.5 mM 2,2′-azino-bis(3-ethylthiazoline-6-sulfonate) (ABTS, Sigma-Aldrich) to its cation radical (ε_436_ = 29,300 M^−1^ cm^−1^) in 100 mM tartrate buffer at pH 3 with 0.1 mM H_2_O_2_.

The versatile peroxidase activity was estimated via the formation of an Mn^3+^ tartrate complex from the direct oxidation of Mn^2+^ (ε_238_ 6500 M^−1^ cm^−1^) using 100 mM sodium tartrate (pH 5) and 0.1 mM MnSO_4_. The assay mixture contained 950 μL substrate, 30 µL 0.1 mM H_2_O_2_, and 50 μL enzyme extract, and was incubated at 24 °C for 1 min [39]. All activities were expressed in international units (IU L^−1^).

### 2.7. Statistical Analysis

The radial growth values and the percentage of decolorization were evaluated via the application of an analysis of variances (ANOVA) and Duncan’s test. The biomass and the enzymatic activity values of *Emmia latemarginata* in submerged fermentation were plotted using the mean, with the error bars representing the standard error of data obtained from triplicate experiments.

## 3. Results

### 3.1. Strains Identification

**Figure 1:**
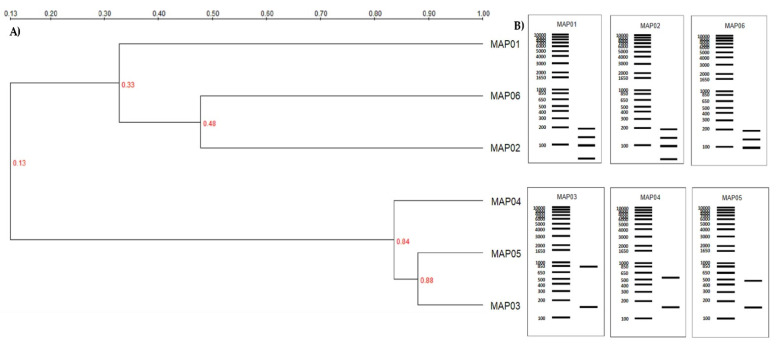
RAPD analysis dendrogram and PCR-RFLP patterns. (**A**) RAPD analysis using Primer 5, (**B**) A PCR-RFLP pattern for the ITS region was performed with two restriction endonucleases on two different strain groups: Group A: *Emmia latemarginata* strains (MAP03, MAP04, and MAP05) used the enzymes *Sal I* and *Alu I*; and Group B: *Mucor circinelloides* strains (MAP01, MAP02 and MAP06) used the enzymes *HindIII* and *MboI*.

Six fungal strains from two main phyla (Basidiomycota and Zygomycota) and two main genera (*Emmia* and *Mucor*) were isolated from a coloured textile industry effluent. The taxonomic classification of each strain was carried out based on the morphological characteristics of the fungi (Figure S1) and via molecular characterization and genetic marker analysis. The RAPD analysis used the three primers discussed above in the methodology section yielded phylogenetic trees formed of two main clusters, in which a different degree of similarity between the fungal isolates can be seen (Figure 1A). The restriction enzymes used in the PCR-RFLP assays were selected by undertaking virtual restriction analysis on the ITS region of the fungal isolates, while the banding profiles also show differences among all the fungal strains (Figure 1B). Both the RAPD and RFLP analyses generated two main strain-related groups: Group A comprised the MAP03, MAP04, and MAP05 strains and, Group B comprised the MAP01, MAP02, and MAP06 strains.

A total of six fungal sequences were amplified, with their length varying between 613 and 709 base pairs (bp). Performed using the BLASTN program [40], the pairwise alignment of the amplified sequences and other related sequences taken from GenBank generated a percentage of identity of between 97.51 and 99.88. BLASTN analysis confirmed that the MAP03, MAP04, and MPA05 sequences were identified as *Emmia latemarginata* with an identity of 97.51–99.67%, while the MAP01, MAP02, and MAP06 strains were identified as *Mucor circinelloides* with an identity 99.67–99.88%.

**Figure 2:**
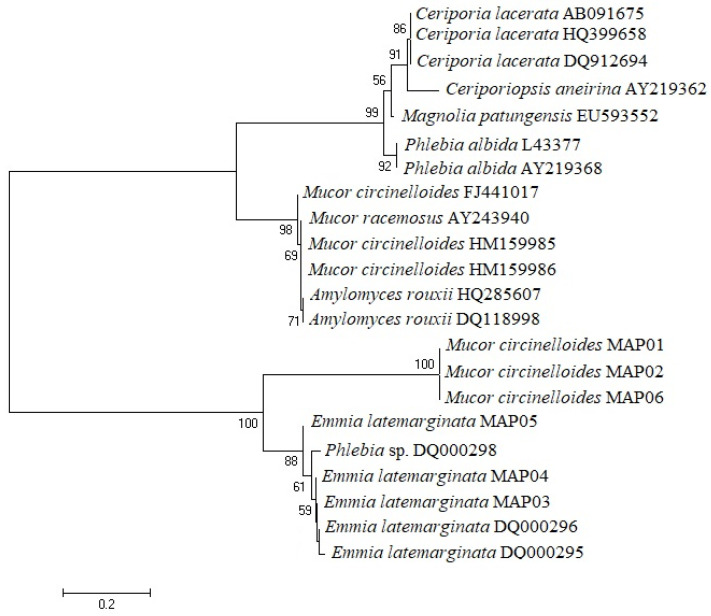
Molecular phylogenetic analysis of fungal isolates. The numbers at the nodes indicate the levels of bootstrap support based on Kimura distance and the neighbour-joining method.

The sequences of *Emmia latemarginata* and *Mucor circinelloides* and the other related sequences obtained from GenBank and examined in the present study presented a length of 527 to 709 bp, while the length of the consensus region for the alignment of all sequences was 1077 bp (Table S1). The phylogenetic tree generated by the sequencing analysis conducted for the ITS region of the rDNA shows that the fungi isolated belong to two different genera, a finding supported by high bootstrap values. The optimal tree with the sum of branch length = 2.28906448 is shown (Figure 2).

### 3.2. Fungal Characterization

#### 3.2.1. Agar-Plate Screening for the Decolorization of Different Dyes

In order to evaluate the decolorization capacity of the fungal isolates, we screened them in seven dyes differing in chemical structure, on agar plates with and without an extra carbon source. Of all the fungal isolates, only the strain identified as *Emmia latemarginata* (MAP03) was able to grow and generate decolorization for Remazol Brilliant Blue R (anthraquinone) and red (azo) dyes on the agar plate without an extra carbon source, although this growth was limited (Figure S2).

**Figure 3:**
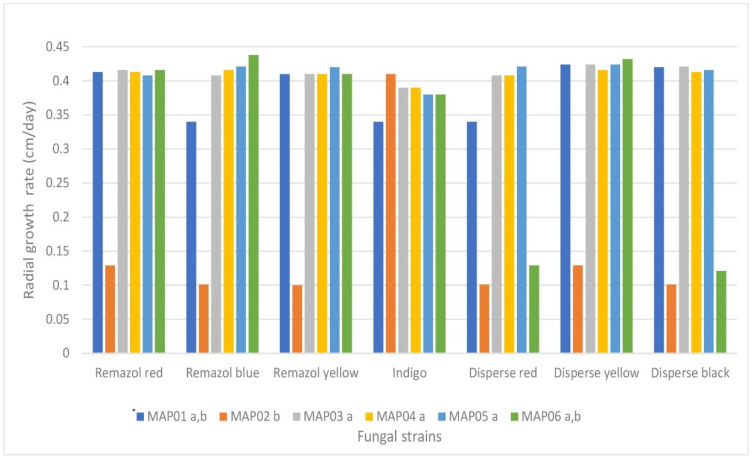
Radial growth rate (cm/day) of MAP strains grown for six days on agar media containing a carbon source and different dyes. * Strains with the same letters (a,b) are not significantly different at a 95% confidence interval.

Figure 3 shows the plate radial growth of the six strains in a medium to which different dyes were added along with starch as a carbon source. All dyes tested were found to have been decolorized, but at different rates. The MAP01, MAP03, and MAP04 strains presented the fastest decolorization rate, while the MAP02 strain did not produce any change in coloration. All the fungi grew in the presence of all of the dyes (*p* < 0.05). However, significant differences were observed among the six strains, wherein the growth rate for the MAP02 strain was the lowest (0.153 cm day^−1^) and the MAP03, MAP04, and MAP05 strains presented the highest growth rates (approximately 0.40 cm day^−1^ or over).

#### 3.2.2. Dye Decolorization in a Carbon-Limited Liquid System

The percentages and rate of decolorization were found to vary among strains and type of dye on a carbon-limited liquid medium (0.3% glucose), with the Remazol yellow and red dyes the least susceptible to decolorization, for all strains, while the indigo, Remazol Brilliant Blue R, and disperse dyes were decolorized by up to 100% for some of the strains (Table 2, Table 3 and Table 4).

The decolorization percentages for indigo (Table 2) ranged from 8 to 100%, while the highest decolorization percentage (100%) was obtained for the MAP03 strain after eight days of fermentation, followed by MAP01 (75%) and MAP04 (66%) after 16 fermentation days. The remaining strains showed variable decolorization percentages that ranged from 8% to 33% after 24 fermentation days.

Table 3 shows the decolorization percentages for the Remazol dyes tested, wherein Remazol blue and yellow were, for all strains, the most and least susceptible, respectively, with average decolorization percentages of 43%, 37%, and 24%, for Remazol blue, red, and yellow, respectively. The highest decolorization percentage (100%) for Remazol blue was obtained with both the MAP05 and MAP03 strains after 24 days of fermentation, followed by MAP04 (73%), as obtained over the same fermentation period. The MAP06 strain was unable to decolorize this dye, presenting only 12% decolorization after 24 days.

For Remazol red and yellow, the highest decolorization percentages were 37% and 33%, respectively, and were obtained using the MAP06 and MAP04 strains, respectively, after 24 days of fermentation. The remaining strains presented decolorization percentages of between 2% and 25% at different times during the fermentation period applied for both dyes.

Table 4 shows the decolorization percentages for the disperse dyes. After 24 days, the average decolorization obtained for all dyes was 63%. However, differences were observed among the strains in terms of the decolorization percentages and fermentation periods for the different dyes. For the disperse red dye, the MAP03 strain presented the highest decolorization percentage (75%) after 24 days of fermentation, followed by MAP02, MAP04, and MAP06 (69%), MAP05 (61%), and MAP01 (47%). The highest decolorization percentages were achieved with the MAP05 strain for disperse yellow (80%) and disperse black (88%) after 24 days of fermentation, while the MAP02 and MAP03 strains were decolorized by up to 75% for the three dyes subsequent to the 24 days of fermentation applied.

#### 3.2.3. Plate Screening of Lignin-Degrading Enzymes

Enzymatic assays were performed directly on MEA plates, with the results obtained from the analysis conducted on all the fungal isolates summarized in Table 5. All strains were positive for peroxidase, while the *Emmia latemarginata* strains (MAP03, MAP04, and MAP05) were also positive for laccase and the *Mucor circinelloides* strains (MAP01, MAP02, and MAP06) were positive for tyrosinase. Phenol oxidase activity was not detected using tannic acid for any of the strains.

### 3.3. Characterization of the Growth and Ligninolytic Enzyme Production of the MAP03 Strain in Submerged Fermentation under Different Conditions

**Figure 4:**
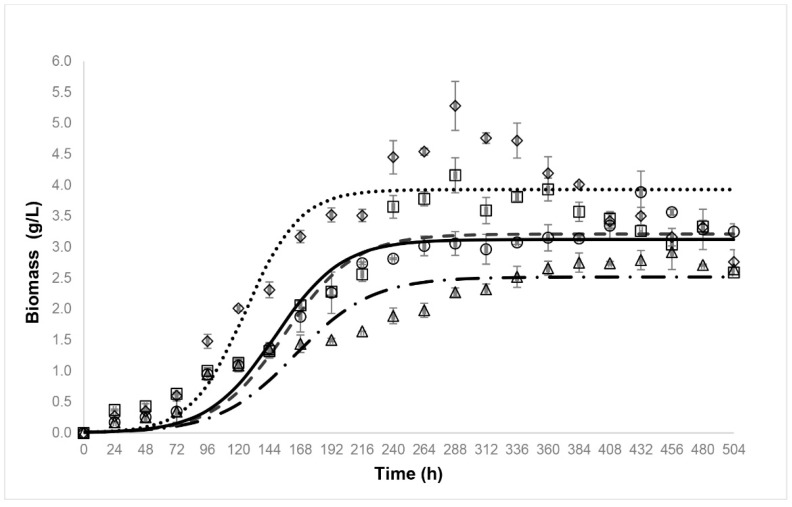
Growth kinetics of *Emmia latemarginata. Emmia latemarginata* was grown in submerged fermentation at 30 °C for 22 days in a basal medium and in the presence of 500 ppm AYG dye, 10% *v/v* wheat straw extract or the combination of both, **○---** Basal medium, **Δ -·-·-** Basal medium + AGY, **◊^….^** Basal medium + wheat straw and **□—** Basal medium + wheat straw + AYG. The error bars represent the standard deviation for the three different fermentation runs.

**Figure 5:**
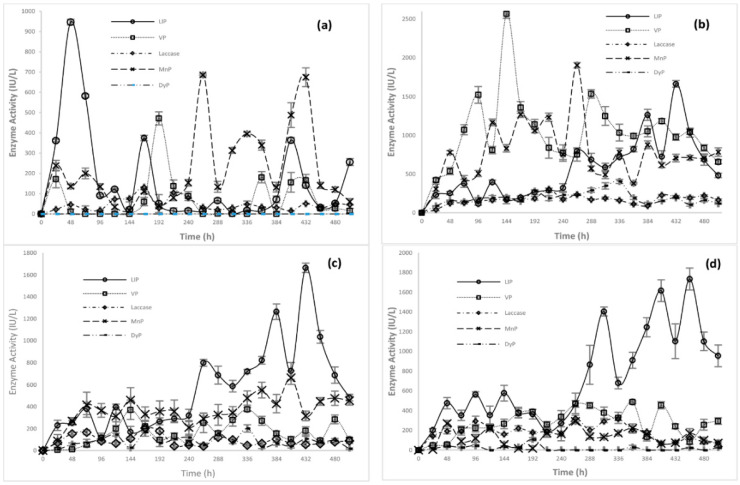
Effect of AYG dye and wheat straw extract on ligninolytic enzyme activity profiles. Time course for the extracellular ligninolytic enzyme activities of *Emmia latemarginata* as obtained in submerged fermentation conditions with (**a**) Basal medium, (**b**) Basal medium + AYG, (**c**) Basal medium + wheat straw, and (**d**) Basal medium + wheat straw + AYG. The error bars represent the standard deviation for the three different fermentation runs.

Given its decolorization capacity, the *Emmia latermaginata* (MAP03) strain was selected for further characterization, wherein we analyzed the effect of the AYG dye, wheat straw extract, and the combination of both on growth and the ligninolytic enzyme activity profile over the time-course of a submerged *Emmia latemarginata* fermentation. Under these conditions, the addition of AYG dye did not affect the maximum production of biomass (*X_max_*) or the specific growth rate (μ). However, the addition of the wheat straw extract increased both kinetic parameters (Figure 4). Moreover, the addition of the AYG dye, wheat straw extract, and the combination of both all produced a clear induction effect on the activity of all the ligninolytic enzymes identified. The main types of enzyme activity detected were VP, MnP, and LiP, with DyP and Lac also detected but at low levels (Figure 5). The addition of AYG dye did not just induce the production of ligninolytic enzymes, but additionally modified the enzyme activity profiles, and maximal activities of VP and MnP were detected in the exponential and beginning of the stationary growth phase and LiP at the end of the stationary phase, in contrast with the maximal activities recorded for these enzymes in the basal medium in which the main enzyme activity recorded was for LiP at the beginning of the exponential growth phase. These results indicate that VP and MnP may play a more significant role than LiP during the decolorization of AYG (Figure 4 and Figure 5).

## 4. Discussion

While dyes and pigments are used in diverse commercial applications, innovative and sustainable effluent treatment processes must be implemented to reduce their environmental impact. Despite the fact that dyes are stable and difficult to degrade, the capacity of fungi to degrade such pollutants has been widely studied and their potential for use in the bioremediation of dye wastewater represents an opportunity not only for resource recovery, but also for environmental sustainability [2,11,41,42].

The present research isolated, from a colored textile industry effluent, fungi that naturally occur in said effluent and have the potential to degrade textile dyes. Six fungal strains from two main phylum (basidiomycota and zygomycota) and two main genera (*Emmia* and *Mucor*) were isolated.

In order to evaluate the decolorization capacity of the fungal isolates, we screened them in seven structurally different dyes. All dyes were decolorized to a different extent both on agar plates with and without an extra carbon source and in liquid fermentation with a limited concentration of carbon source. Among the azo dyes, Remazol yellow and red (mono azo) with a very complex structure and low redox potential were decolorized less efficiently than the rest of the dyes. Both dyes present a naphthalene ring, in addition Remazol yellow presents different functional groups and aromatic rings which make it even more difficult to degrade. Contrarily, indigo (indigoid) and Remazol blue (anthraquinone) were the most susceptible to decolorization, with both dyes having a high redox potential and a less complex structure. It has been reported that the degradation of dyes, which involves aromatic cleavage, depends on the type, number, and position of functional groups and other factors, such as electron distribution and charge density [43,44,45]. Consequently, color removal is less efficient with highly substituted and higher molecular weight dyes as observed in our results.

Furthermore, the addition of a carbon source increased the decolorization capacity for all strains. It has been demonstrated that, in the degradation of dyes and other types of pollutants, the co-metabolism plays an important role in initial microorganism growth. Therefore, the microorganisms do not depend on those compounds as carbon and energy sources [41,46]. Comparing our results for the decolorization percentages obtained on both agar plate and in liquid media, decolorization was more efficient in liquid media, as has been previously demonstrated [47]. The MAP03, MAP04, and MAP05 strains identified as *Emmia latemarginata* were found to be the most efficient in terms of the rate of decolorization and the number of dyes that they were able to decolorize. However, to achieve, higher decolorization efficiencies, it will be necessary to optimize the fermentation conditions, such as pH, temperature, and concentration of carbon source, and more importantly, to optimize these parameters in the presence of different percentages of a real industrial effluent.

Various studies have shown that the efficiency of synthetic dye decolorization depends on both the kind of microorganism used and the dye structure [48,49,50,51,52]. Among the mechanisms that fungi utilize during the decolorization process are adsorption, enzymatic degradation, or a combination of both [15]. Our results clearly show dye adsorption during the first hours of growth, after which the decolorization process was observed to be underway. The use of fungi as biosorbents has been recently proposed as an effective alternative for the treatment of industrial wastewaters [2,42], while Riegas-Villalobos et al. [53] demonstrated the efficient removal of the Orange II dye by *Trametes versicolor* by means of biomass absorption and laccase enzyme oxidation. Wang and Yu [49] showed that mycelial adsorption is one of the most common decolorization effects exerted by the hyphae of *T. versicolor*, a process occurring during the first hours of contact with the colorant, while the level of adsorption depends directly on the source of the dye. Fu and Viraraghavan [54] showed that the decolorization carried out by live cells involves complex mechanisms such as bio-adsorption, and that intra and extracellular oxidases participate in this kind of cellular process, which directly depends on the corresponding nutritional requirements and both the concentration and toxicity of the effluent.

When correlating the lignin-degrading enzymes detected for each strain with the dye decolorization, it was observed that the peroxidase can be one of the main enzymes involved in the decolorization for all strains, while laccase and tyrosinase could also be participating in the process for *Emmia latemarginata* and *Mucor circinelloides* strains respectively.

The ability of filamentous fungi to degrade toxic compounds including dyes has been previously reported [18,55,56]. Several filamentous fungi have been shown to oxidize dyes via peroxidases, phenoloxidases, and laccase [57,58,59]. In addition, *Emmia latemaginata* is known to produce low or null levels of Lac and, depending on the dye, different levels of MnP, VP, and LiP [55,60,61]. Some studies have also explored the potential of *Mucor circinelloides* as a dye degrader [62,63].

The growth kinetics and ligninolytic enzyme activity profile of *Emmia latermaginata* (MAP03) strain in submerged fermentation showed the inductive effect of both AYG dye and wheat-straw extract on the activity of all the ligninolytic enzymes identified. This effect was particularly important for VP followed by MnP.

Our results coincide with reports of compounds derived from agro-industrial residues, such as wheat straw, rice straw, corn stubble, and cane bagasse, functioning as inducers of the main lignin-modifying enzymes in white rot fungi [64,65,66]. Furthermore, the addition of dyes had a clear induction effect on enzymatic activity [67,68]. Our research group has reported the ability of this fungus to decolorize AYG dye [61], while there have also been recent reports of its ability to decolorize both RBBR and Orange G dyes efficiently [55]. However, these are the first reports of analysis being conducted on the ligninolytic enzyme system of *Emmia latemarginata* in submerged fermentation under three different conditions.

## 5. Conclusions

The present study found that the six fungal strains analysed presented a high capacity for decolorizing azo, indigo, and anthraquinone dyes. All the strains studied were able to co-metabolize the dyes of interest in the presence of starch or glucose as an additional carbon source, growing and in turn generating dye decolorization. Two of the Remazol dyes, red and yellow, were less susceptible to decolorization for all the strains used, while the indigo and Remazol Brilliant Blue R dyes were the most susceptible, presenting decolorization percentages of up to 100% for the MAP03 strain. The white rot fungi *Emmia latemargina* (MAP03, MAP04, and MAP05) strains were found to be the most efficient in terms of decolorization rate and the number of dyes they were able to decolorize. Plate screening for lignin-degrading enzymes showed that all strains were positive for peroxidase. In the presence of AYG dye and wheat straw extract, the *Emmia latemarginata* strain (MAP03) produced the highest levels of VP, MnP, and LiP.

Research on new fungal strains capable of efficiently degrading organic dyes remains necessary for applications in the field with real textile industry effluents. The fungal strains analyzed in the present research were isolated from the textile industry effluent and, given the dye decolorization results presented here, could be candidates for further biotechnological research on the decontamination of industrial effluents. Fungi is a great potential option for the degradation of dyes. However, efficient bioremediation must generate less-toxic, inert, or fully degraded compounds. Therefore, further research must be conducted on the products generated by these degradation processes, specifically on their level of toxicity, prior to the practical application of mycoremediation methods.

## Figures and Tables

**Table 1:** Characteristics of the dyes used in the present study.

Colour Index Name	Chemical Structure	Chemical Class	λ_Max_ (nm)	Dye Content (%)	Solubility
Indigo	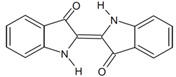	Indigoid	298	96	Insoluble
Remazol (reactive) Yellow145	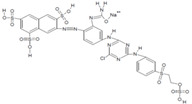	Azo	300		Soluble in H_2_O 80 g/L
Remazol (reactive) Red3B	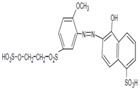	Azo	500	100	No data available
Remazol (reactive) Brilliant Blue R	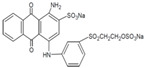	Anthraquinone	600	50	1 mg/mL H_2_O
Disperse Black 1	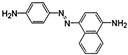	Azo	300		79.3 mg/L H_2_O_2_ soluble in EtOH
Disperse Yellow 3	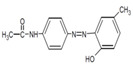	Azo	400	30	1 mg/mL formic acid:95% EtOH(1:1)
Disperse Red 19	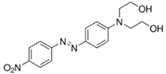	Azo	495	97	0.01 g/L in 50% EtOH
Acetyl yellow G (AYG)	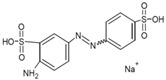	Azo	390	95	No data available

**Table 2:** Percentage of indigo dye decolorization by isolated fungi cultured in a shake flask with carbon-limited medium containing 150 mg L^−1^ dye.

Strain	Fermentation Period (Days)			
	8	16	24	*
MAPO1	Ng	75.8	75.8	^b,c,d^
MAPO2	16.6	25.0	25.0	^b,c,d,e^
MAPO3	100.0	100.0	100.0	^a^
MAPO4	59.1	66.6	66.6	^a,b,c^
MAPO5	31.6	33.3	33.3	^c,d,e^
MAPO6	Ng	8.3	8.3	^e^

* Duncan analysis. Strains with the same letters (a–e) are not significantly different at a 95% confidence interval. Ng = No growth.

**Table 3:** Percentage of Remazol dyes decolorization by isolated fungi cultured in a shake flask with carbon-limited medium containing 150 mg L^−1^ dye.

Strains	Remazol Brilliant Blue R				Remazol Red				Remazol Yellow			
	Fermentation Period (Days)											
	8	16	24	*	8	16	24	*	8	16	24	*
MAPO1	35.9	38.5	45.1	^c^	25.0	25.0	24.5	^a,b^	0.0	10.6	15.5	^b^
MAPO2	Ng	3.5	42.9	^d^	22.5	27.5	24.7	^a,b^	0.0	2.6	20.0	^b^
MAPO3	73.2	82.0	100.0	^b^	25.0	25.0	22.5	^a,b^	6.6	6.6	15.5	^b^
MAPO4	38.5	43.4	73.2	^b,c^	25.0	20.0	25.0	^a,b^	22.2	28.8	33.3	^a^
MAPO5	74.1	95.6	100.0	^a^	2.50	22.5	25.0	^b^	Ng	20.0	10.6	^b^
MAPO6	Ng	Ng	12.2	^d^	27.50	35.0	37.0	^a^	2.2	6.6	11.1	^b^

* Duncan analysis. Strains with the same letters (a–c) are not significantly different at a 95% confidence interval. Ng = No growth.

**Table 4:** Percentage of disperse-dye decolorization with isolated fungi cultured in a shake flask with carbon-limited medium containing 150 mg L^−1^ dye.

Strains	Disperse Red				Disperse Yellow				Disperse Black			
	Fermentation Period (Days)											
	8	16	24	*	8	16	24	*	8	16	24	*
MAPO1	58.3	44.1	47.2	^b^	2.5	12.5	60.0	^a,b^	27.8	32.3	50.0	^b,c^
MAPO2	40.5	41.6	69.4	^a,b^	2.5	12.5	75.0	^a,b^	0.0	7.6	69.2	^b,c^
MAPO3	64.7	65.2	75.0	^a,b^	2.5	12.5	68.7	^a,b^	25.7	45.3	65.3	^a,b,c^
MAPO4	61.1	66.6	69.7	^a,b^	2.5	10.0	45.2	^b^	30.7	31.5	42.6	^b,c^
MAPO5	58.8	58.3	61.1	^a,b^	2.5	27.5	80.0	^a^	28.0	42.3	88.4	^a,b^
MAPO6	15.5	58.3	69.2	^b^	2.5	5.0	47.5	^b^	Ng	Ng	Ng	^c^

* Duncan analysis. Strains with the same letters (a–c) are not significantly different at a 95% confidence interval. Ng = No growth.

**Table 5:** Qualitative assay for lignin-degrading enzymes detection on MEA plates. Oxidative activity was determined on the 7th day of cultivation by adding the specific substrate for each enzyme.

Strains	Laccase		Phenoloxidase	Tyrosinase	Peroxidase
	Guaiacol	O-ANISIDINE	Tannic ACID	*p*-CRESOL	Pyrogallol and H_2_O_2_
MAPO1	−	−	−	+	+
MAPO2	−	−	−	+	+
MAPO3	+	+	−	−	+
MAPO4	+	+	−	−	+
MAPO5	+	+	−	−	+
MAPO6	−	−	−	+	+

Positive reaction +, negative reaction −.

## Supplementary Materials

The following are available online at https://www.mdpi.com/article/10.3390/jof7100805/s1, Figure S1: Morphology of fungal isolated. Figure S2: Growth of *E. latemarginata* (MAP03) on agar to which Remazol dyes without an extra carbon source were added. Table S1: GenBank and culture collection accession numbers of strains considered for the phylogenetic analysis.
