# Supplementary material for: Isolation of Fungi from a Textile Industry Effluent and the Screening of Their Potential to Degrade Industrial Dyes
Source: J Fungi (Basel). 2021 Sep 27;7(10):805. doi: 10.3390/jof7100805 (PMC8540792; doi:10.3390/jof7100805)
Supplement: Supplementary file 1 [file jof-07-00805-s001.zip › jof-1385594-supplementary.pdf]

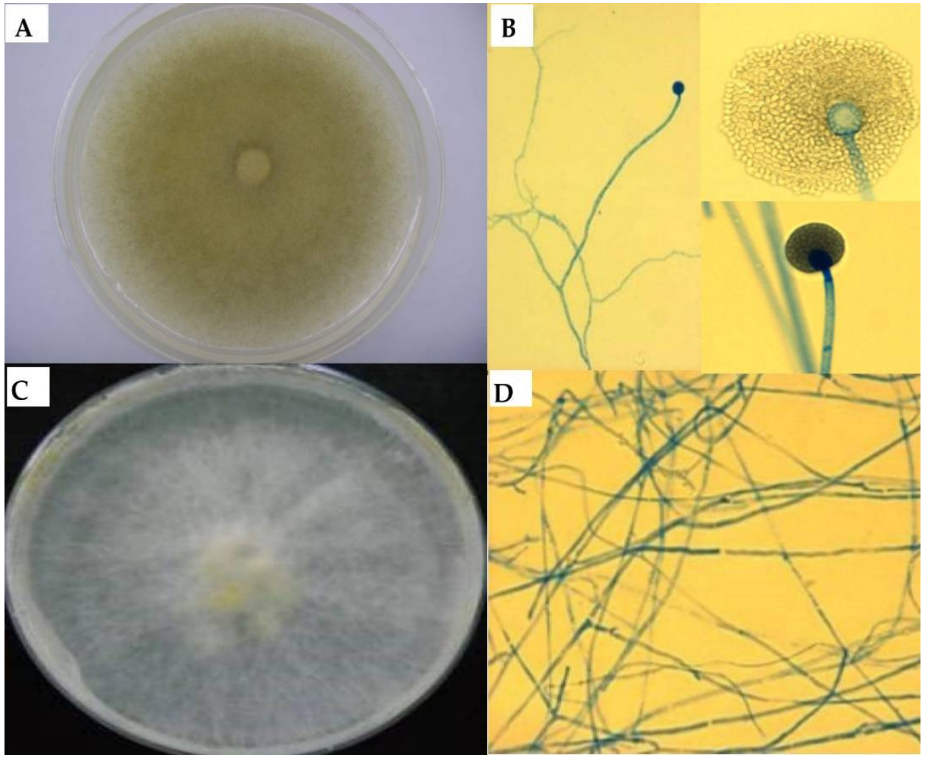


Figure S1: Morphology of fungal isolated. *Mucor circinelloides*: (A) Growth on PDA after 7 days at 30 °C. (B) Microscopic view showing some reproductive structures under 40X . *Emmia latemarginata*: (C) Growth on PDA after 7 days at 30 °C. (D) Microscopic view showing no spore formation, under 40X.

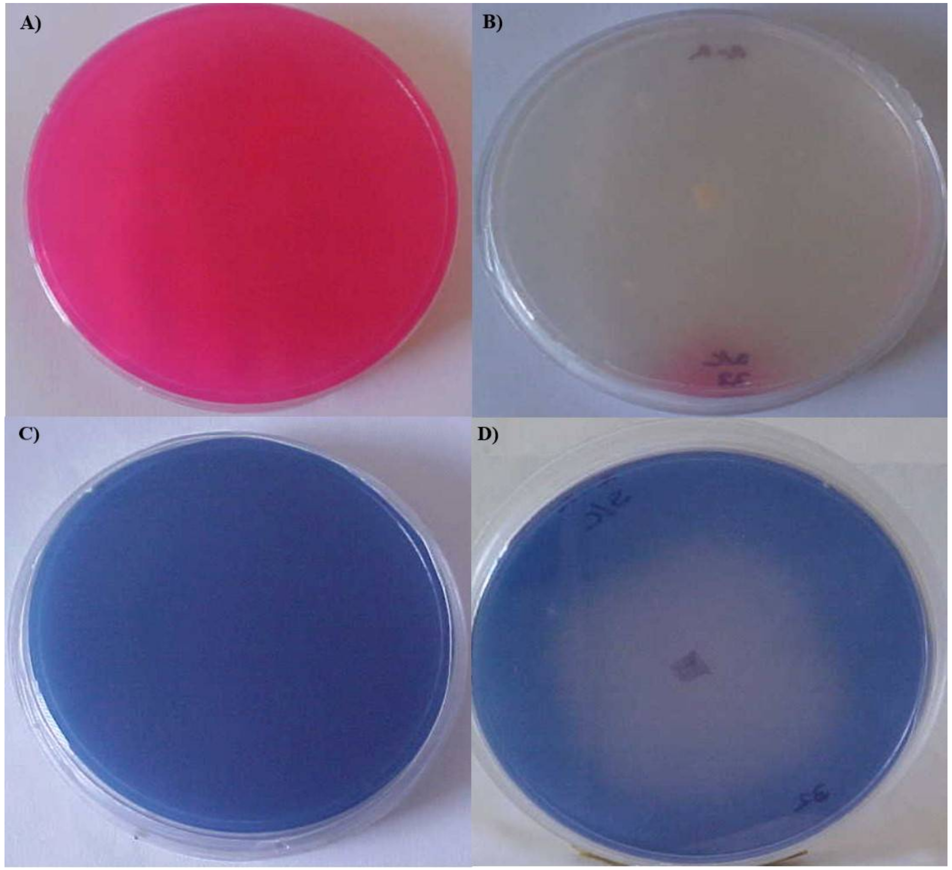


Figure S2: Growth of *E. latemarginata* (MAP03) on agar to which Remazol dyes without an extra carbon source were added. Remazol red: Control (A). The colony after five days of growth at 28 °C (B). Remazol brilliant blue R: Control (C). The colony after five days of growth at 28 °C (D).

**Table S1.** GenBank and culture collection accession numbers of strains considered for the phylogenetic analysis.

| Species | Strain no. | Country | Host | GenBank ITS accession no. | References |
|---|---|---|---|---|---|
| *Phlebia albida* | GB 1833 | Spain | *Pinus* sp. | L43377 | Nakasone, K.K. 1996. Morphological and molecular studies on *Auriculariopsis albomellea* and *Phlebia albida* and reassessment of *A. ampla*. Mycologia. 88: 762-775. |
| *Phlebia albida* | GB-1833 | Spain | *Pinus* sp. | AY219368 | de Koker, T.H., Nakasone, K.K., Haarhof, J., Burdsall, H.H., Janse, B.J.H. 2003. Phylogenetic relationships of the genus *Phanerochaete* inferred from the internal transcribed spacer region. Mycol. Res. 107(PT 9): 1032-1040. |
| *Manglietia patungensis* | ___ | China | ___ | EU593552 | He, S.C., and Ma, L.Y. 2008. The phylogenetic status of *Magnolia wufengensis* and its relationship with other subgen. *Yulania* species. Consulted [14/09/2021]. On line: https://www.ncbi.nlm.nih.gov/nuccore/EU593552 |
| *Ceriporia lacerata* (= *Emmia lacerata*) | KUC3018 | Korea | Treated wood | DQ912694 | Kim, J.J., kang, S.M., Choi, Y.S., Kim, G.H. 2007. Microfungi potentially disfiguring CCA- treated wood. Int. Biodeterior. Biodegradation. 60(3): 197-201. |
| *Ceriporia lacerata* | SFFPS MZ-340 | Japan | ___ | AB091675 | Suhara, H., Daikoku, C., Takata, H., Suzuki, S., Matsufuji, Y., Sakai, K., Kondo, R. 2003. Monitoring of white-rot fungus during bioremediation of polychlorinated dioxin-contaminated fly ash. Appl. Microbiol. Biotechnol. 62 (5-6): 601-607. |
| *Ceriporia lacerata* | HJF085 | South Korea | Old books | HQ399658 | Park, J. H., Kim, Y. H., Kim, Y. S., Kim, H. J., Kim, T. J. 2010. Microorganisms found on old books in Sungkok library of Kookmin University. Direct submission. Consulted [14/09/2021]. On line: https://www.ncbi.nlm.nih.gov/nuccore/hq399658 |
| *Ceriporiopsis aneirina* | FP-104462-sp | USA | *Populus tremuloides* | AY219362 | de Koker, T. H., Nakasone, K. K., Haarhof, J., Burdsall, H. H., Janse, B. J. 2003. Phylogenetic relationships of the genus *Phanerochaete* inferred from the internal transcribed spacer region. Mycol. Res. 107 (PT 9): 1032-1040. |

| | | | | | |
|---|---|---|---|---|---|
| *Emmia latemarginata* (= *Oxyporus latemarginata*) | MAP04 | Mexico | Textile industry effluent | ___ | This study |
| *Emmia latemarginata* | MAP05 | Mexico | Textile industry effluent | ___ | This study |
| *Emmia latemarginata* | MAP03 | Mexico | Textile industry effluent | ___ | This study |
| *Emmia latemarginata* | CTM10136 | Tunisia | ___ | DQ000296 | Dhouib, A., Hamza, M., Zouari, H., Mechichi, T., Hmidi, R., Labat, M., Martinez, M. J., Sayadi, S. 2005. Screening for ligninolytic enzyme production by diverse fungi from Tunisia. World J. Microbiol. Biotechnol. 21(8-9): 1415-1423. |
| *Emmia latemarginata* | CTM10133 | Tunisia | ___ | DQ000295 | Dhouib, A., Hamza, M., Zouari, H., Mechichi, T., Hmidi, R., Labat, M., Martinez, M. J., Sayadi, S. 2005. Screening for ligninolytic enzyme production by diverse fungi from Tunisia. World J. Microbiol. Biotechnol. 21(8-9): 1415-1423. |
| *Phlebia* sp. | CTM10125 | Tunisia | ___ | DQ000298 | Dhouib, A., Hamza, M., Zouari, H., Mechichi, T., Hmidi, R., Labat, M., Martinez, M. J., Sayadi, S. 2005. Screening for ligninolytic enzyme production by diverse fungi from Tunisia. World J. Microbiol. Biotechnol. 21(8-9): 1415-1423. |
| *Mucor circinelloides* | MAP06 | Mexico | Textile industry effluent | ___ | This study |
| *Mucor circinelloides* | MAP01 | Mexico | Textile industry effluent | ___ | This study |
| *Mucor circinelloides* | MAP02 | Mexico | Textile industry effluent | ___ | This study |

| | | | | | |
|---|---|---|---|---|---|
| *Mucor circinelloides* | dx-29 | China | Mushroom | FJ441017 | Sun, H., Song, R., Deng, X. 2008. Fungi isolated from mushroom. Direct Submission. Consulted [14/09/2021]. On line: https://www.ncbi.nlm.nih.gov/nuccore/fj441017 |
| *Mucor circinelloides* | OTU31 | China | Soil | HM159986 | Liu, J., Shen, D. 2010. GenBank Direct Submission. Consulted [14/09/2021]. On line: https://www.ncbi.nlm.nih.gov/nuccore/hm159986 |
| *Mucor circinelloides* | OTU30 | China | Soil | HM159985 | Liu, J., Shen, D. 2010. GenBank Direct Submission. Consulted [14/09/2021]. On line: https://www.ncbi.nlm.nih.gov/nuccore/hm159985 |
| *Mucor racemosus* | CBS 111561 | Netherlands | Sufu pehtze | AY243940 | Han, B. Z., Kuijpers, A., Thanh, N., Nout, M. 2003. Mucoraceous molds involved in the commercial fermentation of sufu pehtze. Direct Submission. Consulted [14/09/2021]. On line: https://www.ncbi.nlm.nih.gov/nuccore/ay243940 |
| *Amylomyces rouxii* (= *Mucor rouxii*) | KCCM60146 | Korea | ___ | HQ285607 | Yang, S., Lee, Y.W. 2010. Fungal Species Associated with Traditional Starter Cultures (Nuruks) Used for Rice Wine in Korea. Consulted [14/09/2021]. On line: https://www.ncbi.nlm.nih.gov/nuccore/hq285607 |
| *Amylomyces rouxii* | CBS 416.77 | France | Infected tissues | DQ118998 | Schwarz, P., Bretagne, S., Gantier, J.C., Garcia-Hermoso, D., Lortholary, O., Dromer, F., Dannaoui, E. 2006. Molecular identification of zygomycetes from culture and experimentally infected tissues. J. Clin. Microbiol. 44(2): 340-349. |

Sequences under study used for multiple alignment:

```
>1Emmia.latemarginataMAP03
      1 gtagagtcac ctgatttgag ctagattgtc ataaattgtc tcggtaagag acgactataa
     61 gcatgaacta ataaatactt caacaccaca gcgcagataa ttatcacact gaaggcgatc
    121 cgtaagattc acgctaatgc atttcagagg agtcgactag aagccgacac aacctccaag
    181 tccaagccca ctaaacttca ttacaaaatt taggggttga gaattccatg agactcaaac
    241 aggcatactc ctcggaatac caaggagtgc aaggtgcgtt caaagattcg atgattcact
    301 gaattctgca attcacatta cttatcgcat ttcgctgcgt tcttcatcga tgcgagagcc
    361 aagagatccg ttgctgaaag ttgtatataa ttgtgttata cacagtaaac attctataac
    421 tgaagcgttt gtagtaaaca taagaaaggc ttattaccaa ctattaaata gctggcttac
    481 accgtttctt acataaagtg cacagaggtt gagagtggat gagccaggtg tgcacatgcc
    541 tcgttaaagg ccagctacaa cccgttcaaa actcgataat gatccttccg caggttcacc
    601 tacggaaacc ttgttctctt acttacttcc gatggggtat tgcggagatt taagaggggg
    661 gaggaggaat aaaaggcagg gcggagggct ggccgcgggc gaagagacac acgcgaggga
```

```
      721 gggaagggag gaggggaagt aagaagaaga ggaaaaggga aaaggaaaag agaagaaaag
      781 caggggggaac acaggagaag agaaagacac ataaggtagg agagtggaga ggaagcaaag
      841 aggagacaaa gagaatacaa gaaaagatga aagaaagaga taagggaaga caaggaatac
      901 agaagcgtga gagagaagag gagagcgaca cgaagaggag gcatagtaag ggtaaggaag
      961 aacgggagac aagaaatgca taaaag

>2Emmia.latemarginataMAP04
        1 gtgatagtgt ccacctgatt tgagctcaga ttgtcataaa ttgtctcggt aagagacgac
       61 tataagcatg aactaataaa tacttcaaca ccacagcgca gataattatc acactgaagg
      121 cgatccgtaa gattcacgct aatgcatttc agaggagtcg actagaagcc gacacaacct
      181 ccaagtccaa gcccactaaa cttcattaca aaatttaggg gttgagaatt ccatgagact
      241 caaacaggca tactcctcgg aataccaagg agtgcaaggt gcgttcaaag attcgatgat
      301 tcactgaatt ctgcaattca cattacttat cgcatttcgc tgcgttcttc atcgatgcga
      361 gagccaagag atccgttgct gaaagttgta tataattgtg ttatacacag taaacattct
      421 ataactgaag cgtttgtagt aaacataaga aaggcttatt accaactatt aaatagctgg
      481 cttacaccgt ttcttacata aagtgcacag aggttgagag tggatgagcc aggtgtgcac
      541 atgcctcgtt aaaggccagc tacaacccgt tcaaaactcg ataatgatcc ttccgcaggt
      601 tcacctacgg aaaccttgtt ctcgtgagaa cttcctgttt ttcatagata gagaaatcaa
      661 agtcaataaa gtgatagaga tcagggata

>3Mucor.circinelloidesMAP06
        1 gggcatccgc ctgatttaga tcaatttaaa aaaagtatta tttgggaggc cccagcacag
       61 tttaccgcaa gagcttctct ttatattaaa aaaaagttca ggcattcaaa caagatcagg
      121 cctttgtaca tttcaagagg ttcgagatca gaatagatca agagactctc agtattccta
      181 ttcaacaaaa tgttggatag agggtttgtt ttgatactga aacaggcgtg ctcattggaa
      241 taccaatgag cgcaagttgc gttcaaagac tcgatgattc actgaatatg caattcacac
      301 tagttatcgc actttgctac gttcttcatc gatgcgagaa ccaagagatc cgttgttaaa
      361 agttgtttta tagatttttt aggtctatgt tacaatatta attctgaatt cttttggtaa
      421 ataataatag gataccaagc ctaagcttga ttatgactcg gttagcatct ccatcgccta
      481 tccttatagc agtggagcat ccctcaagcg tcaagtaata atacagttca cagtaaatag
      541 ataataatgg acaagccaaa attattgatt atttaatgat ccttccgcag gttcacctac
      601 ggaaaccttg ttca

>4Mucor.circinelloidesMAP02
        1 gatcgtttcc cgcctgattt cgatcaattt aaaaaaagta ttatttggga ggccccagca
       61 cagtttaccg caagagcttc tctttatatt aaaaaaaagt tcaggcattc aaacaagatc
      121 aggcctttgt acatttcaag aggttcgaga tcagaataga tcaagagact ctcagtattc
      181 ctattcaaca aaatgttgga tagagggttt gttttgatac tgaaacaggc gtgctcattg
      241 gaataccaat gagcgcaagt tgcgttcaaa gactcgatga ttcactgaat atgcaattca
      301 cactagttat cgcactttgc tacgttcttc atcgatgcga gaaccaagag atccgttgtt
      361 aaaagttgtt ttatagattt tttaggtcta tgttacaata ttaattctga attcttttgg
      421 taaataataa taggatacca agcctaagct tgattatgac tcggttagca tctccatcgc
      481 ctatccttat agcagtggag catccctcaa gcgtcaagta ataatacagt tcacagtaaa
      541 tagataataa tggacaagcc aaaattattg attatttaat gatccttccg caggttcacc
      601 tacggaaacc ttattcgttt ttttttttc tacca
```

```
>5Mucor.circinelloidesMAP01
        1 tggattccgc ctgatttcga tcaatttaaa aaaagtatta tttgggaggc cccagcacag
       61 tttaccgcaa gagcttctct ttatattaaa aaaaagttca ggcattcaaa caagatcagg
      121 cctttgtaca tttcaagagg ttcgagatca gaatagatca agagactctc agtattccta
      181 ttcaacaaaa tgttggatag agggtttgtt ttgatactga aacaggcgtg ctcattggaa
      241 taccaatgag cgcaagttgc gttcaaagac tcgatgattc actgaatatg caattcacac
      301 tagttatcgc actttgctac gttcttcatc gatgcgagaa ccaagagatc cgttgttaaa
      361 agttgtttta tagatttttt aggtctatgt tacaatatta attctgaatt cttttggtaa
      421 ataataatag gataccaagc ctaagcttga ttatgactcg gttagcatct ccatcgccta
      481 tccttatagc agtggagcat ccctcaagcg tcaagtaata atacagttca cagtaaatag
      541 ataataatgg acaagccaaa attattgatt atttaatgat ccttccgcag gttcacctac
      601 ggaaaccttg tctttttttt tttttcatca caa

>6Emmia.latemarginataMAP05
        1 ggcagtcttc ctgatttcga gctcagattg tcataaattc gtctcggtaa gagacgacta
       61 taagcatgaa ctaataaata cttcaacacc acagcgcaga taattatcac actgaaggcg
      121 atccgtaaga ttcacgctaa tgcatttcag aggagtcgac tagaagccga cacaacctcc
      181 aagtccaagc ccactaaact tcattacaaa atttaggggt tgagaattcc atgagactca
      241 aacaggcata ctcctcggaa taccaaggag tgcaaggtgc gttcaaagat tcgatgattc
      301 actgaattct gcaattcaca ttacttatcg catttcgctg cgttcttcat cgatgcgaga
      361 gccaagagat ccgttgctga aagttgtata taattgtgtt atacacagta aacattctat
      421 aactgaagcg tttgtagtaa acataagaaa ggcttattac caactattaa atagctggct
      481 tacccgtttt taataagtgc acagaggttg agagtggatg agccagtgtg cacatgcctc
      541 gttaaaggca gctacaaccc gttcaaactc gataatgatc cttcccaggt ccctacgaaa
      601 cttgtacatt ttttttttaa
```

NOTE: The six MAP fungal sequences analyzed in this study are currently in the process of being published in the NCBI GenBank database under the following Submission number: **SUB10380912**
